# Designing Ecological Security Patterns Based on the Framework of Ecological Quality and Ecological Sensitivity: A Case Study of Jianghan Plain, China

**DOI:** 10.3390/ijerph18168383

**Published:** 2021-08-08

**Authors:** Xueping Su, Yong Zhou, Qing Li

**Affiliations:** 1 The College of Urban & Environmental Sciences, Central China Normal University, Wuhan 430079, China; suxueping0504@mails.ccnu.edu.cn; 2 Key Laboratory for Geographical Process Analysis & Simulation of Hubei Province, Central China Normal University, Wuhan 430079, China

**Keywords:** ecological quality, ecological sensitivity, ecological security pattern, ecological corridor, circuit theory

## Abstract

Researchers and managers of natural resource conservation have increasingly emphasized the importance of maintaining a connected network of important ecological patches to mitigate landscape fragmentation, reduce the decline of biodiversity, and sustain ecological services. This research aimed to guide landscape management and decision-making by developing an evaluation framework to construct ecological security patterns. Taking the Jianghan Plain as the study area, we identified key ecological sources by overlaying the spatial patterns of ecological quality (biodiversity, carbon storage, and water yield) and ecological sensitivity (habitat sensitivity, soil erosion sensitivity, and water sensitivity) using the Integrated Valuation of Environmental Services and Tradeoffs (InVEST) model and the Chinese Soil Loss Equation Function. Ecological corridors were obtained by the least-cost path analysis method and circuit theory. A total of 48 ecological sources (3812.95 km^2^), primarily consisting of water area, forestland, and cropland, were identified. Ninety-one ecological corridors were derived, with a total length of 2036.28 km. Forty barriers and 40 pinch points with the highest improvement coefficient scores or priority scores were selected. There were 11 priority corridors with very high levels of connectivity improvement potential and conservation priority, occupying 16.15% of the total length of corridors. The overall potential for ecological connectivity is high on the Jianghan Plain. Our framework offers a valuable reference for constructing ecological security patterns and identifying sites for ecological restoration at the regional scale.

## 1. Introduction

Over the last decade, intensified human activities have accelerated land cover transformation, which has increased land fragmentation, disrupted ecological processes, and caused declines in local biodiversity, ecological services, and ecological environment [1]. Under this background, governments worldwide have taken action in ecological restoration and conservation [2], including China [3]. In 2019, the Ministry of Natural Resources of the People’s Republic of China issued a nationwide territory development plan (TDP), which aims to improve people’s quality of life and achieve the sustainable development of society by reasonably planning the locations of agricultural production space, urban living space, and ecological space [4]. One of the critical components of the TDP is constructing ecological security patterns (ESPs) [5].

Research on ESPs has come from global studies on ecological networks (ENs), especially from analyses of ENs/greenways in Europe [6] and America [7]. The frameworks for ESP and EN studies are similar—‘ecological sources (ESs)—ecological resistance surfaces—ecological corridors’ [8]. Research on ESPs helps ecological conservation by identifying important regional areas and critical areas for ecological restoration [5]. Different research backgrounds (e.g., economic status, technical resources, social development status) of EN and ESP studies have led to differences in the understanding of, the evaluation methods used for identifying ecological sources, and the construction of ecological resistance surfaces and ecological corridors. However, in general, EN/ESP studies have been widely recognized due to their contribution to regional health and sustainable development [9]. Studies on ENs in Europe focused on animal conservation [10,11,12,13,14,15,16]. The common step is as follows: first, researchers located the potential habitats as ecological sources by identifying migration species’ characteristics, especially how the studied species migrated, mated, and found food. Then, researchers chose indicators and parameters for resistance surfaces based on the preferred habitat and behavioral preference of the studied species. Finally, the researchers simulated the migration route of targeted species by analyzing ESs and resistance surfaces of the research area. For example, G. Modica et al. (2021) considered 66 terrestrial faunal focal specie, assessed the habitat quality for each focal faunal species, and mapped the overall habitat quality. Then, they identified the habitat patches and considered the minimum foraging requirements of each focal species and the possibility of animal movement among different patches. According to percolation theory directional and least-cost connectivity analysis, they finally implemented multispecies ecological networks at Calabria [11].

In America, EN/greenway design combines multiple objectives, such as recreational and leisure services, historical and cultural preservation, and ecological protection. Greenways enhance residents’ quality of life by contributing to physical health and exercise [17,18,19]) and providing activity-promoting transportation opportunities that link urban parks and neighborhoods [20]. Regardless of their design and structure, greenways represent “multiple objective, open space corridors that perform natural functions while offering desirable aesthetic qualities to humans as they recreate or commute along trails” [20,21,22]. Therefore, ecological corridor planning considers the accessibility of ESs and ecological corridors and then designates areas that are close to people, have connectivity, and provide ecological services [7].

Constrained by the concentration of a growing population in urban systems, in China, the construction of ESPs aims to enhance the supply of urban ecosystem services [23] by connecting important ecological areas (mountains, rivers, forests, farmlands, lakes, grasslands, etc.) to ensure the integrity of the structure and function of the ecosystem, which are significant for national and regional ecological security [5]. Ecosystem services comprise ‘the ecosystems conditions or processes utilized, actively or passively, to produce human well-being’ [24]. Ecosystem services have clear promise to help identify and protect priority areas for biodiversity [25,26]. Mapping techniques have provided a powerful tool for integrating complex information related to ecosystem services into landscape management and environmental decision-making [27,28,29]. Normally, the ecological service system is comprised of three key areas, including the service providing area, service connecting area, and service benefiting area [30,31]. Ecological sources in ESPs studies are similar with the concept of the service providing area but are not totally the same. In the study of ESPs, scholars define ecological sources as places that are important in the ecosystem and provide humans with abundant ecological services or places that are more vulnerable or sensitive to human activities or natural disasters [5,32,33]. The ecological corridor in ESP research is defined as an ecological corridor that connects the ecological sources [34,35,36,37,38]. The construction of ecological corridors in ESPs aims to help maintain ecological services, sustain ecological processes, and accelerate species migration [9]. An ESP is a complete EN that can provide ecosystem services with a rational ecosystem structure for the well-being and quality of life of growing populations and for economic and social development. ESPs are important component of national security [39].

In conclusion, unlike EN or greenway research, which focuses only on targeted migrating species (European mode) or focuses on providing ecological corridors for recreation (U.S.A. mode). ESPs in China identify ecologically important areas (high in ecological services) and ecologically sensible/vulnerable areas and connect them to maintain and improve ecological services to sustain the development of cities [32].

There are many methods for identifying ecological sources, assigning values to ecological resistance surfaces, and extracting ecological corridors.

There are two main approaches used for ES selection. One method is based on land-cover classification, and the other is multi-indicator ecological evaluation [16,40]. Identifying ESs by land-cover type is efficient, simple, but coarse because it does not pay attention to ecological processes, functions, and interactions with the landscape matrix [41]. With the development of society and technology, observation data and field data have become abundant. Multi-indicator evaluations of ecological services and of the ecological sensitivity of land are based on the spatial characteristics of the study area emerged. The indicators selected for ecological evaluation are mainly derived by spatial overlay or model assessment [33]. For example, on the biodiversity function of land, Gao Mengwen et al. (2021) reclassified the slope and vegetation coverage rate and merged them to represent the biodiversity status in the study area [39]. Gao Yang et al. (2020) used the results from the habitat quality model to indicate the biodiversity function [42]. There are also many different assessment methods used to determine water yield and the carbon storage function of land [43]. The accuracy of the results by spatial overlay is relatively low compared with the results derived by model assessment, but spatial overlay methods need fewer data than does model assessment. Researchers normally select evaluation frameworks and indicators according to regional characteristics. For example, according to the ecological problems in Fengxian County (e.g., aggravation of soil and water losses, land desertification and land salinization), Jin et al. (2021) selected soil and water loss sensitivity, desertification sensitivity in plain areas, and salinization sensitivity as the indicators for evaluating ecological sensitivity [44].

Regarding assigning values to ecological resistance surfaces, the most common approach in EN studies is to assign resistance values to different land-cover types based on the migration habits of the targeted species in the study area [11]. However, this is not the case for research on ESPs in popular dense areas in China. Due to the limited allocation of research funds, detailed studies on animal migration habits are rare in urban agglomerations in eastern and central China. Biodiversity in eastern and central China has declined because governments are more focused on economic development [45]. There has been little financial support for wildlife research, and few research teams have conducted detailed surveys of species migration pathways and pattern characteristics in densely populated areas [46,47]. However, without ecological corridors, habitats in densely populated regions remain fragmented. If the status quo is maintained, the ecological function and biodiversity of the area will continue to decline. In this case, when setting the parameter for the resistance surface, researchers who have studied ESPs in densely populated regions have usually derived the parameter by referring to ESP studies on areas with similar climate and natural resources or by assuming parameters based on general animal migration habits [37,38,48,49,50]. Assuming that most animals are sensitive and avoid being close to urban land, a higher resistance value is given to urban land, and a lower resistance value is given to wetlands, grassland, and forest. This assignment is consistent with reality. For example, the nocturnal migration behavior of whooper swans (Cygnus cygnus) is to avoid disturbance by human activity during the day. The probability of wildlife occurrence decreases as the amount of human activity decreases [51]. Birds tend to be close to wetlands/forests because there is less human activity in protected wetlands/forests, and the vegetation in these areas provides food and habitat [52]. However, land-cover types do not reflect all attributes of the land. Based on this shortcoming, Tong et al. (2020) used nighttime lighting data to modify the ecological resistance surface assigned by land-cover classification [53]. In addition to giving ecological resistance to surfaces solely based on land-cover cover types or modified land-cover types, there is a multi-indicator assessment approach [53]. Another commonly used evaluation framework for constructing ecological resistance surfaces is to evaluate ecological resistance from three aspects: topography (e.g., slope, altitude), eco-environment (e.g., distance from water bodies, land-cover cover types), and threats from human society (e.g., distance from roads, distance to pollution sources) [54]. The ecological resistance surfaces derived by this evaluation framework are more accurate than those derived solely by land-cover cover type.

Widely used analytical approaches for deriving ecological corridors include least-cost paths (LCPs) [55], circuit theory [56], and graph theory [57,58]. Each approach is designed to meet different objectives and will produce different outcomes. The LCP is the most traditional and commonly used approach, which contiguously collects cells with the lowest cumulative value as the path crosses from one endpoint to the other endpoint. As LCP assumes that individuals have perfect knowledge of the landscape and therefore select a single optimal route, the results of LCP may not be the choice of animals in reality [59,60]. In this case, McRae et al. (2008) combined circuit theory from physics with landscape ecology, assuming that the migration process of species in the landscape has electron-like properties, i.e., random flow in a circuit, to identify multiple pathways in the underlying landscape [61]. Circuit theory is based on random-walk theory and results in an implicit assumption that individuals moving across a landscape do not know the relative resistance beyond their immediate surroundings [62]. Graph theory modeling uses habitat patches as points of connection and links as corridors. This approach could be combined with LCP modeling or circuit theory to derive corridors and habitat patches [63]. In conclusion, the circuit model more accurately approximates how individuals move through real landscapes. This model is useful for evaluating connectivity and identifying constrained areas (bottlenecks) for possible conservation action [64].

**Figure 1:**
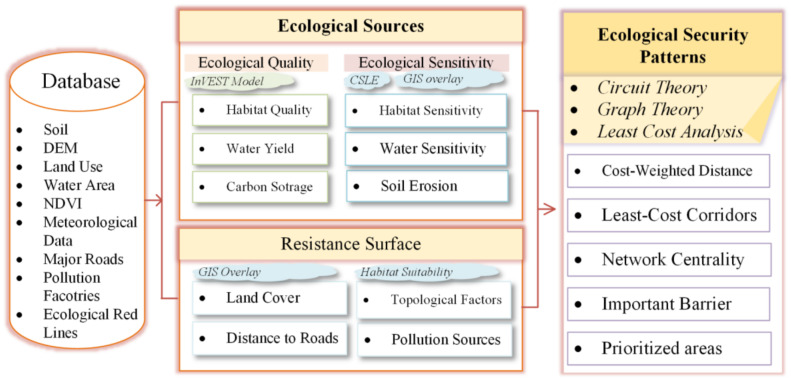
Research framework.

Recently, research on ESPs has mainly used counties or cities as the research area. These studies are practical because they can be integrated into the regional spatial planning of that county or city. However, these studies have not considered the integration of ecosystems. For example, if one of the ESs in county A is in the radiation of one of the ESs in county B, those studies failed to derive. Therefore, the study of ESPs is better focused on a complete physical geographic landscape than on an administrative unit. The primary objective of our study was to present a comprehensive approach framework for constructing ESPs and identifying key points for ecological conservation and ecological restoration. In this study, we calculated the resistance surface of the research area based on the characteristics of wildlife on the Jianghan Plain by referring to other studies (Figure 1). Then, we derived ecological corridors between each ecological source on the Jianghan Plain based on least-cost analysis and circuit theory using Circuitscape 4.1. We identified the important barriers for improving habitat connectivity, determined which areas should be prioritized, and calculated the network centrality of ESs and corridors. The combination of graph theory modeling and circuit theory modeling could quantify the potential corridors and habitat patches that may help land managers to prioritize units that should receive more protection [56,65]. This objective was partitioned into four subobjectives: (1) identify the key ecological space in the Jianghan Plain based on the assessment of ecological quality and ecological sensibility, (2) model potential corridors and construct ESPs and prioritize corridors according to their relative importance and the effect of each corridor on global connectivity, (3) identify the key locations where conserving ecological functions and improving ecological connectivity should be conducted, and (4) assess the robustness. The novelty of this study lies in that it not only synthesizes important and targeted ecosystem services and ecological sensitivity indicators at the regional scale to systematically assess ecological status and construct an ESP but also considers which ecological corridors and sites should be better protected or where it is necessary for a site’s ecological functions to be restored to improve landscape connectivity [29].

## 2. Materials and Methods

### 2.1. Study Area and Data Sources

#### 2.1.1. Study Area

**Figure 2:**
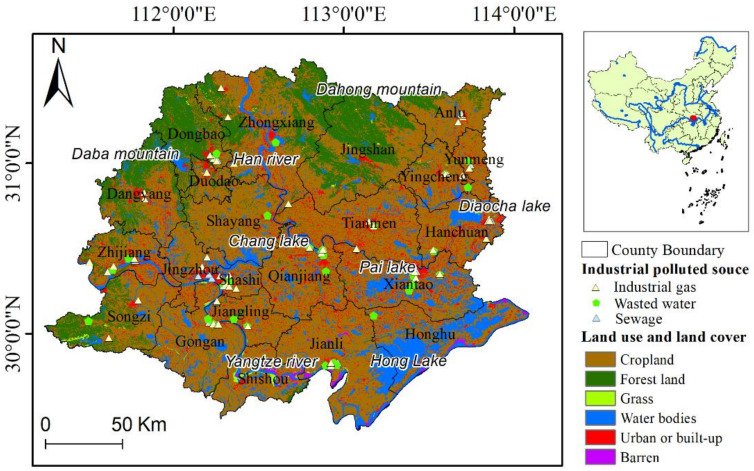
Land use and land cover in the Jianghan Plain.

Jianghan Plain comprises 22 counties (Figure 2). The total area is 41,828.28 km^2^. The study area covers mainly cropland, accounting for 61.75% of its total area. The rest of the land use areas are forestland (16.26% of the total area), water bodies (13.27%), urban or built-up (7.95%), grassland (0.36%), and barren land (0.41%). Forestland is mainly distributed in Daba Mountain in Dangyang County and Dongdao County and Dahong Mountain in northern Jingshan County and Zhongxiang County.

The Jianghan Plain, with its dense network of rivers, was a complete wetland ecosystem in prehistoric times, but over the past 2000 years, under the influence of human activities, its natural wetland landscape gradually transformed into a farmland landscape. In the 13th century, the river was cleared of wetlands and forest swamps, and the dominant waterfowl populations were traditionally geese and cranes. At that time, waterfowl (feathers), ivory, and rhinoceros skin (leather) were the main economic resources of Chu. At the end of the 17th century, the final abolition of the “river and lake office”, a fishing tax institution in the Jianghan Plain, marked the decline in river, lake, and wetland areas due to the cultivation of lakes and fields. Short-tailed albatross (*Diomedea albatrus*) and spotted-billed pelican (*Pelecanus roseus*) were once widely distributed here before the mid-19th century but are now nearly extinct [66,67,68].

Existing common animals include birds such as the white stork (*Ciconia ciconia*), black stork (*Ciconia nigra*), great bustard (*Otis tarda*), Reeves’ pheasant (*Syrmaticus reevesii*), owls (*Strigiformes*), black-faced spoonbill (*Platalea minor*) [69]; mammals such as the dhole (*Cuon alpinus*), porcupine, and hog badger (*Arctonyx collaris*) [70]; and amphibians such as the Chinese giant salamander (*Andrias davidianus*) and great tree frog (*Zhangixalus dennysi*) [71]. The behavioral activities of these animals are sensitive to the quality of human activities and the natural environment. Therefore, the trade-off between economic development and ecological conservation requires a sustainable spatial plan for natural resource management.

#### 2.1.2. Data Sources

The data sources of this paper are described in Table 1. The ecological red line of Hubei is the protected natural area in Hubei.

### 2.2. Research Framework

#### 2.2.1. Identifying Ecological Sources

While ecosystem services are often provided by large natural ecosystems (mountains, forests, rivers, etc.), they are also supplied in urban ecosystems [53]. The goal of ESPs is to increase ecological connectivity and optimize land use/landcover (LULC) arrangements in selected region which can maximise the local supply of ecosystem ser-vices [77]. ESs are an important part of ESPs because they are starting points or destinations of organisms, materials, energy, and information flow [35]. 

Based on the notions of ecological connectivity [29], multifunctionality of ecosystems and maximization of benefits for both humans and natural conservation [78,79,80], we synthesized the complex ecological problems and specific ecological significance of metrics using three key ecosystem services and three ecological sensitivity indicators [35,79,80]. These indicators are popular in ecosystem assessment research [81]. Habitat, the key element of ecology, is related to biodiversity. The higher the quality of the habitat is, the more biodiversity can be preserved. Habitat quality directly influences ecological functions and ecological services, impacting multiple ecological processes, e.g., pollination and nutrient cycles [13,82]. Landscape fragmentation in the Jianghan Plain is serious due to rapid urban sprawl, which poses a threat to wildlife habitat [83]. As one of the important regulatory functions of terrestrial ecosystems, carbon sequestration plays a substantial role in climate regulation [84]. The Jianghan Plain is in the north-south climate transition zone, with ample rainfall, sunshine, and heat resources. There are abundant wetlands, forests, grasslands, and water bodies [85]. Therefore, land cover with high carbon sequestration ability should be better protected [86]. Water is an essential part of life. After the implementation of the South-to-North Water Diversion Project, the total amount of water released from the Danjiangkou Reservoir decreased, and the infrastructure at the main diversion gates and river diversions in the middle and lower reaches of the Han River deteriorated, reducing the water supply for agricultural production [87,88]. Water yield is also an important regulative ecological service on the Jianghan Plain. In conclusion, we took habitat quality, carbon storage, and water yield as three indicators as the main eco-system services and quantified them by the InVEST model.

Ecological sensitivity is another principal factor for identifying potential eco-environmental problems. Natural reserves are sensitive and vulnerable to high-intensity human activity. Ecological red lines are the primary category of protected regions in China, providing individuals and populations with natural resources, habitats and ecological services. By the end of 2018, 40 national nature reserves and 39 provincial natural reserves and other important wildlife habitats (e.g., forest parks, wetlands) were widely distributed in the Jianghan Plain [89]. In addition, water is a fundamental resource for regional economic and social development, and a water sensitivity assessment is required because of the double effect of waste and pollution [90]. Furthermore, soil erosion is a severe problem affected by heavy precipitation during spring and summer and the high intensity of construction. Therefore, we selected three ecological sensitivity indicators: habitat sensitivity, water sensitivity, and soil erosion sensitivity. The three indicators were computed using GIS spatial overlap analysis and the Chinese soil loss equation (CSLE).

We normalized the integrated ecological service and ecological sensitivity by the max-min method, gave them equal weight, and combined them to obtain the ecological importance map. Then, we considered the ecological red line (the national protected reserves). Given the lower radiation and weak connectivity of small patches [50], the source patches with an area less than 10 km^2^ were removed from ecological sources

#### 2.2.2. Ecological Services

##### Habitat Quality

Habitat quality in the InVEST model refers to the ability of the landscape to pro-vide conditions appropriate for individual and population persistence. The model em-phasizes landscape diversity and the corresponding landscape quality. This approach analyzes the relative effect of each threat, the relative sensitivity of each habitat type to a threat, the distance between habitats and threat sources, and the degree to which the land is legally protected [91]. The habitat quality score ranges from 0 to 1. A higher re-gional habitat quality value will receive a higher score.

By referring to previous research [83], we chose urban, rural residential, other con-struction land, arable land, main roads, and railroads as the anthropogenic threat fac-tors of habitats. Additionally, we selected arable land, forest, grassland, water bodies, and unused land as natural habitats for different creatures. The parameters for the model are provided in Appendix A (Table A1). The impact *i_rxy_* of threat *r* from grid cell *y* on the habitat in grid cell *x* is represented by the following equations:

(1) $$i_{rxy}=1-\left(\frac{d_{xy}}{d_{r\max}}\right)\;\mathrm{if}\;\mathrm{linear}$$

(2) $$i_{rxy}=\exp\left(-\left(\frac{2.99}{d_{r\max}}\right)d_{xy}\right)\mathrm{if}\;\mathrm{exponential}$$

where *i*_*rxy*_ is the impact of threat *r* in raster *y* on habitat *x*, *d_xy_* is the linear distance between grid cells *x* and *y*, and *d_r_*_max_ is the maximum effective distance of the threat.

*D_xj_* gives the total threat level in grid cell *x* with land use/land cover (LULC) or habitat type *j* and is calculated as follows:

(3) $$D_{xj}=\sum_{r=1}^{R}\sum_{y=1}^{Yr}\left(\frac{W_{r}}{\sum_{r=1}^{R}W_{r}}\right)r_{y}i_{rxy}\beta_{x}S_{jr}$$

where *D_xj_* is the habitat degradation or total threat level in grid cell *x* with LULC or habitat type *j*, *R* is the number of threat factors, *r* represents the threat layer, and *Y_r_* indicates the set of grid cells on *r*’*s* raster map. *W_r_* indicates the weight of each threat factor (value range from 0 to 1). *r_y_* indicates the effect of threat r that originates in grid cells; *i_rxy_* indicates the distance between habitat and the threat source and the impact of the threat across space; *βx* is the factor that mitigates the impact of threats on habitat by environmental policies (here, *βx* = 1); *S_jr_* indicates the sensitivity of LULC type *j* to threat factor *r*; the weights of threats are normalized so that the sum across all threat weights equals 1. By normalizing weights such that they sum to 1, we can think of *D_xj_* as the weighted average of all threat levels in grid cell *x*. The map of *D_xj_* will change as the set of weights we use changes.

A grid cell’s degradation score is translated into a habitat quality value using a half-saturation function where the user must determine the half-saturation value; furthermore, as a grid cell’s degradation score increases, its habitat quality decreases.

(4) $$Q_{x,y}=H_{j}\left(1-\left(\frac{D_{x,j}^{z}}{D_{x,j}^{z}+K_{}^{z}}\right)\right)$$

where *Q_x,y_* is the quality of habitat in parcel *x* that is in LULC *j*; *H_j_* indicates the habitat suitability of LULC type *j*; *k* is the half-saturation constant; *z* = 2.5. More details of this model can be found in the InVEST user’s guide [91].

##### Water Yield

The freshwater supply is represented as the water yield and derived from the InVEST-Water Yield model. The InVEST-Water Yield model estimates the annual average quantity of water produced by a watershed. The water yield in InVEST is defined as the amount of water lost from the landscape, and it calculates the sum and averages of the water yield based on the principle of water balance at the sub-watershed level [91,92]. The annual water yield *Y* (*x*) for each pixel on landscape *x* is determined as follows:

(5) $$Y(x)=\left(1-\frac{AET(x)}{P(x)}\right)P(x)$$

where *AET*(*x*) is the actual annual evapotranspiration for pixel *x*, and *P*(*x*) is the annual precipitation on pixel *x*. The parameters are presented in Appendix A (Table A1).

##### Carbon Storage

Soil carbon sequestration relates to the climate, plants, and agriculture. The carbon storage model in InVEST uses a land-cover map to estimate the amount of carbon storage in a landscape. The total carbon storage C (t.ha^−1^) equals the sum of the carbon stock in four carbon pools (aboveground biomass, belowground biomass, soil, and dead organic matter) [93,94]. The parameters are presented in Appendix A (Table A1).

### 2.3. Ecological Sensitivity

#### 2.3.1. Habitat Sensibility

Habitat sensibility was derived by overlaying the score of land-cover type, the normalized difference vegetation index (NDVI), distance to major roads, and distance to ecological red lines [93,94] (Table 2). The higher the habitat sensibility place is, the higher the score it will obtain.

#### 2.3.2. Water Sensibility

Water sensibility is affected by the distance to the water area and water pollution source (Table 3) [88,89]. We found the list of wastewater discharge plants, sewage treatment plants, and pollution gas emission plants published online by the Hubei Environmental Protection Bureau and located each plant by Ovitalmap software. The places closer to the water area and pollution source had a higher water sensibility [88].

#### 2.3.3. Soil Erosion Sensitivity

Researchers around the world use the soil erosion index to measure the effectiveness of soil conservation [95]. Soil erosion changes the landscape, disrupts the carbon cycle at multiple scales, and reduces crop yield and biomass production [96]. The higher the soil erosion is, the lower the amount of soil conserved is. The most common models employed for soil erosion assessment are the Soil and Water Assessment Tool (SWAT) model, the Revised Universal Soil Loss Equation (RUSLE) model [95], and the CSLE [97]. Each model has its drawbacks and strengths. The RUSLE model is effective for large-region evaluations [95], and the CSLE is more suitable for soil erosion studies in China [97]. In this study, we used the CSLE as the following equation:

(6) A=R×K×LS×C×P×T

where A is the soil loss in t.ha^−1^ yr^−1^. R is the rainfall erosivity in MJ.mm.ha^−1^.h^−1^.yr^−1^. K is the soil erodibility in t.h. MJ^−1^ mm^−1^. L and S are dimensionless topographic factors of the slope length and the slope steepness, respectively. C is the dimensionless vegetation cover factor of biological practices for trees, shrubs, and grasslands. P is the dimensionless factor of engineering practices. T is the dimensionless factor of tillage practices such as crop rotation, contour tillage, residue cover, and intercropping strips [97].

#### 2.3.4. Integrated Resistance Surface

The resistance surface parameterization in the present study is generally simplified to set the resistance value according to the land-cover cover type [93]. Nevertheless, this method ignores other perturbing factors on animal movement, such as the influence of topography, road traffic, towns, water pollution, and air pollution [39]. By referring to an ESP study on Jiangxi (central Yangtze river) [42] and Jiangxi (lower Yangtze river) [88], the setting of the resistance surface in this paper is shown in Table 4.

#### 2.3.5. Extraction of Ecological Corridors

To find the least-cost corridors between ES patches and the rank of connectivity importance in these corridors, we used linkage pathways to map the least-cost corridors. We used Centrality Mapper to detect the centrality of ES patches and corridors. In the Linkage Pathways tool, each cell in a resistance map is attributed a value reflecting the energetic “cost,” (i.e., difficulty and mortality risk) of moving across that cell. As animals move away from specific core areas, cost-weighted distance analyses produce maps of the total cumulative movement resistance. The optimal corridors and potential corridors were derived in this way. In the Centrality Mapper tool, each core area is treated as a node, and each link is assigned a resistance equal to the cost-weighted distance of the corresponding least-cost corridor. The higher the score of the centrality of a path is, the higher the number of ESs it connects. The higher the centrality of the ecological patch is, the higher the number of adjacent ecological patches and corridors it will influence [64]. In addition, we calculated the improvement score and priority score of each ecological corridor. We found the top 40 important barriers and 40 pinch points in the Jianghan Plain to detect the place where ecological resistance needs to be lowered and the most important area in corridors for ecological conservation. More details on circuit theory and Circuitscape software can be found in McRae et al. (2008) [61] and McRae and Shah (2009) [91].

The width of ecological corridors impacts the ecological function of a corridor, and the width of reasonable corridors varies with species, corridor structure, and connectivity. Csuti et al. (1991) claimed that corridor width is important because edge effects penetrate some distance into the corridor. Typically, edge effects can be measured 200–600 m into a forest from the edge, and corridors narrower than 1200 m will not contain a true interior habitat [98]. The width for the migration of large mammals should be sev-eral hundred meters or more. Harris (1991) noted that when we take all species into account, the appropriate corridor width should be greater than 915 m for assemblages of entire species [99]. Based on published research [100,101], we concluded that the dispersal distance of most terrestrial animals and birds on the Jianghan Plain ranges from 3 to 2000 m. Thus, 1 km should be an appropriate width for most species in the study area. Therefore, the width of ecological corridors in the Jianghan Plain was de-fined as 1 km. Based on this assumption, the minimum search radius of Barrier Mapper was set to 200 m with the same size as the grid, and the maximum search radius was set to 1000 m. The barrier was searched by the moving window method.

We used the zonal statistic as a table function of ArcGIS to extract the average improvement scores of each corridor, and the ecological corridors were divided by the quantile method into levels of connectivity enhancement potential, with the highest connectivity enhancement potential being the very high level and the lowest enhancement potential being the low level.

## 3. Results

### 3.1. Spatial Pattern of Ecological Services, Ecological Sensitivity, and Ecological Sources

**Figure 3:**
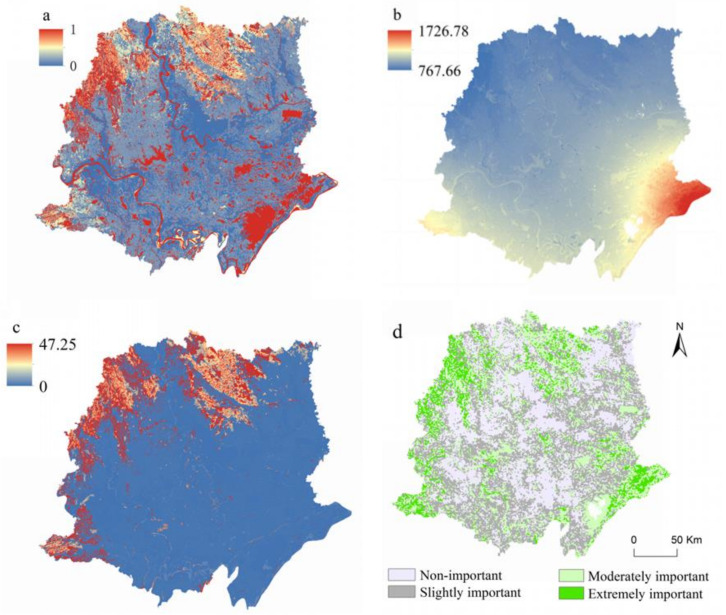
Ecosystem services in the Jianghan Plain. (**a**) Habitat quality (1 represents the place with the highest habitat quality, 0 reflects the place with the lowest habitat quality); (**b**) water yield (mm/yr); (**c**) carbon storage (t.ha^−2^yr^−1^); (**d**) integrated ecosystem service).

The spatial patterns of habitat quality, carbon storage, water yield, and integrated ecological services on the Jianghan Plain are shown in Figure 3. The spatial patterns of these ecological services varied, mainly due to different land-cover types and topologies. The high habitat quality area was in the north of Jianghan Plain where Jin Mountain and Dahong Mountain are; this region has wetlands, lakes, and rivers that are high in habitat quality, such as the Han River in the middle of the study area, the Yangtze river in the southeast and Honghu Lake in the southeast. The low habitat quality area was distributed in the center of the study area where there is intensive human activity, less vegetable coverage, and in more built-up areas. The high carbon storage area was in the north and in the western mountainous area where the vegetation density is high, including Jin Mountain, Julong Mountain, and Dahong Mountain. The low carbon storage area was distributed in the center and south of the study area, where intensive built-up areas, rivers, and lakes are located. The place with the highest level of water yield was in the southeastern Jianghan Plain, which is the lowest area of the Jianghan Plain, and many rivers, including the Han River, Yangtze river, and Dafu River, merge in this area. The integrated ecological service map shows the combined characteristics of the above three ecological services on the Jianghan Plain. We divided the results into four levels (non-important, slightly important, moderately important, and extremely important). The land with the highest level of ecological service, covering 3723.95 km^2^, accounted for 9.08% of the study area. The land-cover types mainly included forestland, cropland, and water bodies, accounting for 49.00%, 25.48%, and 20.83% of the extremely important area, respectively.

**Figure 4:**
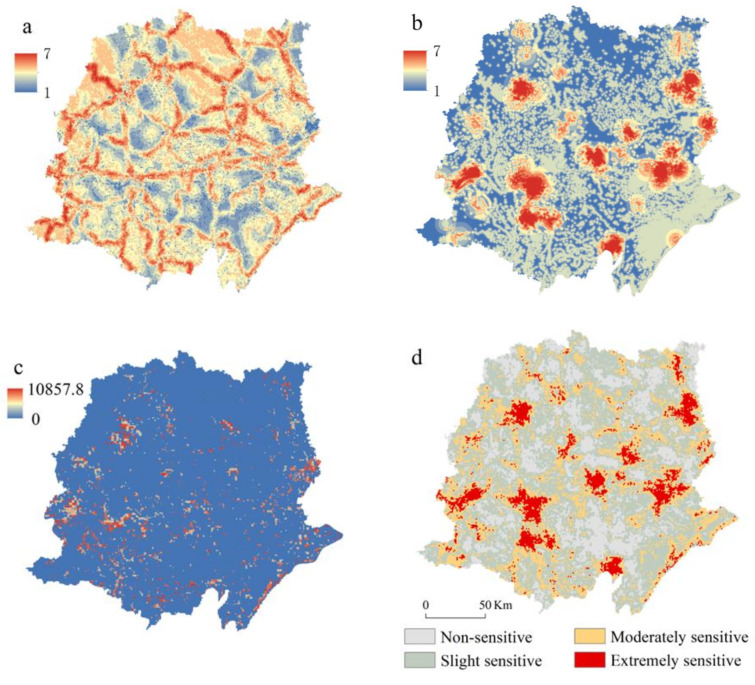
Ecological sensitivity in Jianghan Plain. (**a**) Habitat sensitivity; (**b**) water sensitivity; (**c**) soil erosion sensitivity(t.hm^−2^); (**d**) integrated ecological sensitivity).

The spatial pattern of ecological sensitivity, including habitat sensitivity, water sensitivity, and soil erosion sensitivity, is shown in Figure 4. Places with high soil erosion were mainly distributed at the foot of mountains or along rivers, especially along the lower ridge of rivers. This result was because the human activity in these places was high and the soil cover (C), conservation practices (P), and tillage operations (T) were lower than those in the mountainous area.

Almost every county had a high level of water sensitivity, except for Zhongxiang city and Jinshan city. These two cities had more forestland and less water area than other cities. The ecological red line area (natural reserves) and main roads had high habitat sensitivity. For example, the Hanjiang wetland nature reserve in Jinmen city and the Changhu wetland nature reserve in Jinmen city are vital for rare animals such as the leopard, forest musk, Oriental white stork, and golden eagle. Suizhou Ginkgo Forest Natural Park is an important habitat for ginkgo. The integrated ecological sensitivity map shows that the central urban area was more vulnerable and influenced by multiple factors, including poor soil protection, radiation of pollution sources, and human activity interference. The three indicators derived the comprehensive ecological sensitivity. The result was divided into four levels (non-sensitive, slightly sensitive, moderately sensitive, and extremely sensitive). Extremely sensitive places were clustered at the edges of cities, where polluted industries were located and where there was a high density of roads. The land with extreme sensitivity covered 3232.90 km^2^, accounting for 7.83% of the research area. The land-cover types of the extremely sensitive areas mainly included cropland, built-up area, and water bodies, accounting for 55.11%, 19.56%, and 16.31% of the extremely sensitive area, respectively.

**Figure 5:**
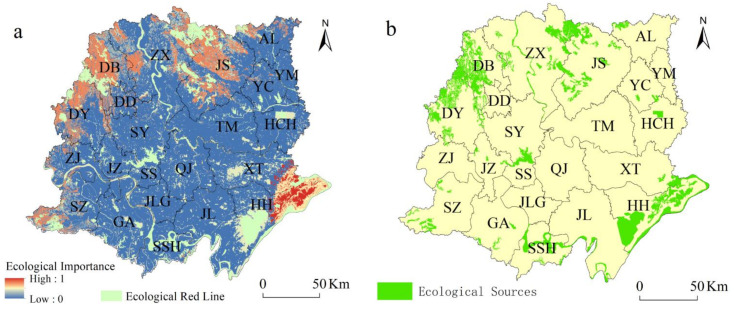
(**a**) Ecological importance and (**b**) ecological sources.

The areas with higher ecological importance (Figure 5a) were distributed in the north and southeastern Jianghan Plain, mainly located in Zhongxiang city, Dangyang city, Dongbao district, Jinmen city, and Songzi city. The less ecologically important areas were mainly in the urban areas of Tianmen, Xiantao, Qianjiang, and Hanchuan.

In this study, 48 ES lands were identified (Figure 5b), totaling 3812.95 km^2^, accounting for 9.12% of the total area of the study area. The ecological red line area (3082.40 km^2^) accounted for 80.84% of the total area of ESs. It mainly consisted of water (1827.12 km^2^), forestland (1755.36 km^2^), and arable land (463.68 km^2^), among which the ecological land area of Honghu city was the largest, amounting to 20.10% of the total area of ESs. However, the ecological land area of Jiangling County was the smallest, amounting to 0.00058% of the total ESs. The ESs that were delineated covered spaces including Yuquansi National Forest Nature Park, Zhangjiahu National Wetland Nature Park, Changhu Ecological Reserve, Yangtze river Chinese sturgeon provincial nature reserve, etc.

### 3.2. Integrated Resistance

**Figure 6:**
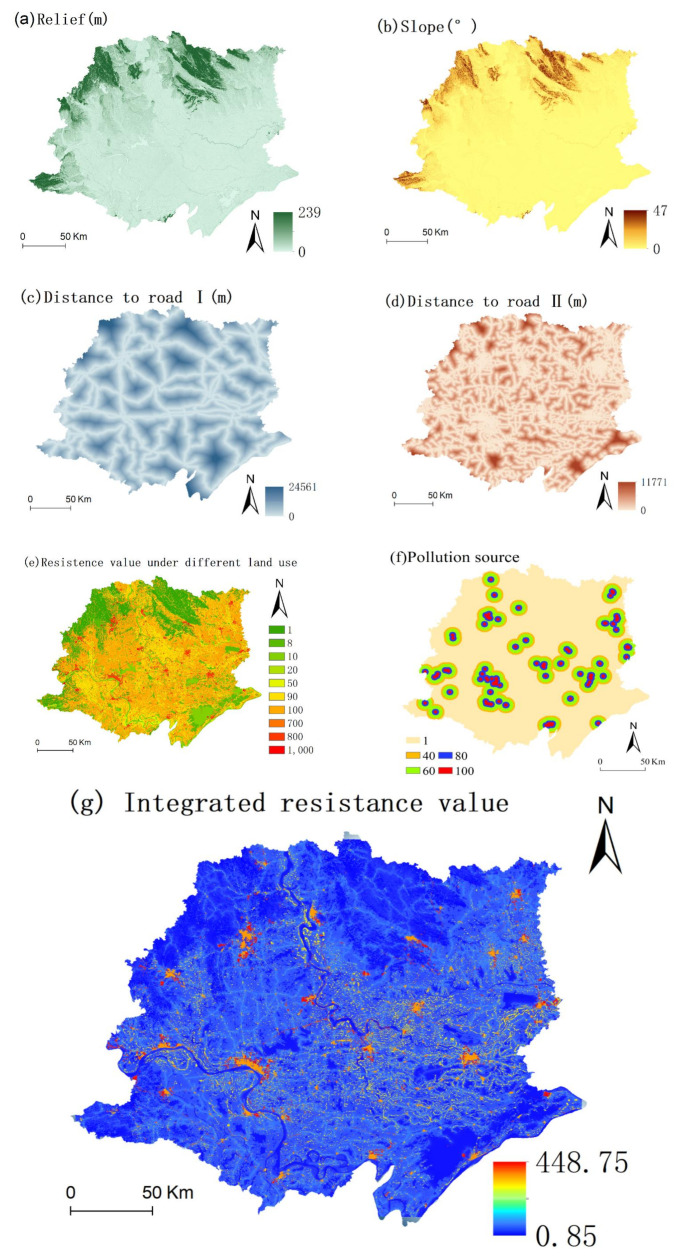
Resistance indicators and integrated resistance surface. (**a**) Degree of relief; (**b**) slope; (**c**) distance from first-class roads; (**d**) distance from second-class roads; (**e**) basic resistance of land-cover types; (**f**) distance to pollution sources; (**g**) integrated resistance value.

The highest resistance value was 441.40, the minimum resistance value was 1, and the average resistance value was 65.24 (Figure 6). The spatial variation in the integrated resistance was high. Generally, places that do not have nonpermeable land but have lower relief, lower slopes, lower distances from roads and fewer pollution sources have low resistance. The integrated resistance map showed that the highest resistance area was in the highly popular density area, mainly in the inner cities. Roads, polluted sources, and other interferences were densely concentrated in cities. The cropland around the cities had the second-highest level of resistance. Although cropland has a high level of vegetation coverage, human management makes it suitable only for a specific crop, which hinders the growth of other species. Thus, the resistance value of cropland was relatively high.

### 3.3. Location of Optimal Ecological Corridors and Potential Corridors

**Figure 7:**
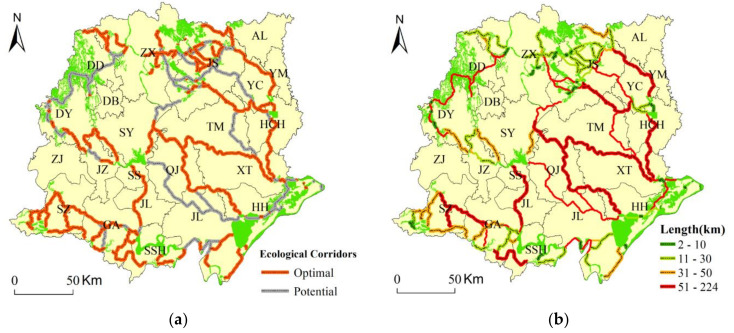
(**a**) Location of optimal ecological corridors and potential corridors and (**b**)length of ecological corridors.

As shown in Figure 7, 91 optimal ecological corridors were derived, and they had a total length of 2036.28 km. A total of 51 potential corridors were derived, with a total length of 3172.48 km, of which 42.26% of the potential corridors overlapped with the optimal corridors. Within these optimal corridors, 38 ecological corridors were less than 10 km in length. The total length of these 38 corridors was 138.90 km, accounting for 6.82% of the total length of the optimal ecological corridors. The shortest corridor was 0.2 km. The corridors that were shorter than 30 km, accounting for 74.73% of the total ecological corridors, were clustered in the northwest, southeast, and north of the study area.

The ESs in the central Jianghan Plain were the hubs in ecological migration in the northwest and southeast, and the corridors from the central to the southeast areas were longer, with most main connecting corridors having lengths longer than 50 km. Thus, the overall spatial characteristics of ecological corridors showed a northwest and southeast direction. The ESs in the north and south of the Jianghan Plain were more complex, with gradually increasing connectivity, forming an EN pattern composed of ESs and corridors.

### 3.4. Centrality of Ecological Corridors and Sources

**Figure 8:**
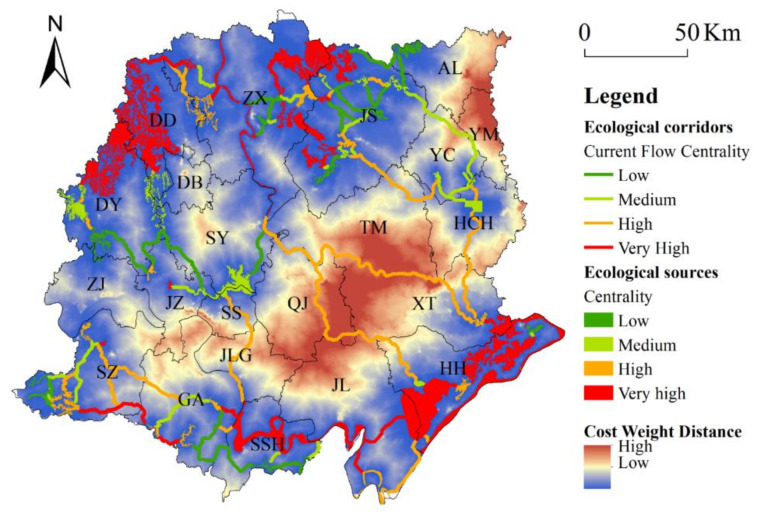
The centrality of habitat corridors and ecological sources.

Current flow centrality indicates how important a corridor or ecological area is for keeping the overall EN connected. The more ESs an ecological corridor/ES connects, the higher centrality it has. Ecological sources with high connectivity were mainly located in the Honghu National Nature Reserve and the Hubei Yangtze river Xinluo Baiji National Nature Reserve in Honghu city (HH), the Hubei Yangtze river Swanzhou Baiji Dolphin National Nature Reserve in Shishou city (SSH), the Dagou Forest Park and Geopark in Zhongxiang city (ZX), the Tiger Claw Mountain Forest Park and the Green Forest Park in Jingshan city (JS), and the Zhanghe National Wetland Nature Park and Xianju River National Wetland Nature Park in Jingzhou (JZ). The ecological corridors with high connectivity mainly connected the ESs from the Han River in Shayang County (SY) to Honghu City (HH), the Shahu Wetland from the Han River to Xiantao city (XT), the Tiger Claw Mountain Forest Park in Jingshan city (JS) to the Lao Guanghu National Wetland Natural Park in Xiaogan (XG), and the ESs from the National Wetland Park in Hanchuan (HCH) to the northern part of Honghu City (HH) (Figure 8).

### 3.5. Locations of Barriers and Priority Area

**Figure 9:**
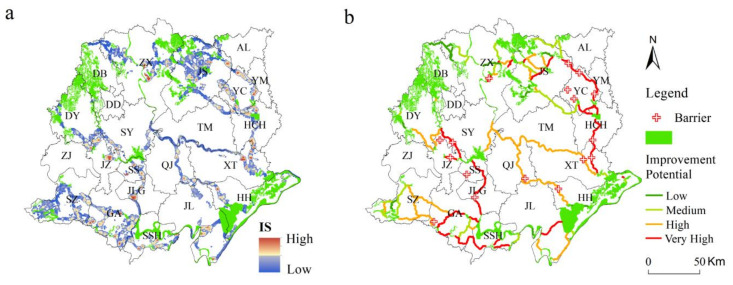
(**a**)Improvement scores (ISs) and barriers of ecological corridors in the Jianghan Plain and (**b**)the improvement potential level of each corridor.

The overall landscape connectivity of the Jianghan Plain region still has more room for improvement, with points of high improvement scores (ISs) for each corridor. By overlaying each obstacle point with the land-cover map, the land types were identified, and the obstacle point land was mainly urban construction building land (15 barriers), industrial and mining land (18 barriers), and arable land (5 barriers) (Figure 9). Due to the gradual shrinkage and narrowing of the lake and river and the problem of pollution, forest resources are decreasing and fragmentation is gradually increasing; these conditions put pressure on the ecological environment and decrease the connectivity between habitats. These conditions change the original flow direction and flow speed of water bodies, thus hindering the migration path of aquatic and terrestrial organisms in the development process to a certain extent and having a negative impact on the vegetation in the region and the formation on both sides of the road. The use of modern agricultural technology has gradually changed the rice crop from mixed to monoculture planting, the current planting rate in the Jianghan Plain has reached 30%, and some of the mechanized planting areas may destroy the habitat connectivity because human activities in such areas tend to be more intensive; additionally, the spatial distribution was mostly located at the connection of ESs and ecological corridors, which was a key location for connectivity, especially in JS, YC, HCH, ZX, XT and other counties.

**Figure 10:**
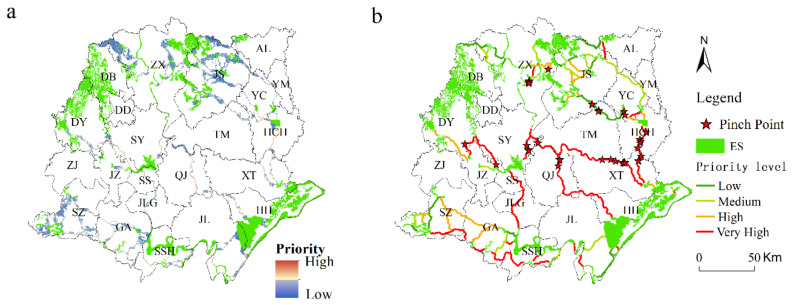
(**a**) Priority and (**b**) pinch points of ecological corridors on the Jianghan Plain.

Pinch points are places where the loss of a small area could disproportionately compromise connectivity. The places that have a higher influence on corridor connectivity would obtain a higher priority score. The pinch points should be protected as priority areas (Figure 10).

In this study, a total of 40 pinch points were identified, whose land-cover types were mainly forestland, water area, and farmland, of which 27 were water areas, 5 were forestland, 8 were farmland, and XT had the most, with 9 pinch points, of which 2 pinch points were farmland, and 7 were water areas. The next highest number was in HCH, with 7 pinch points, of which 2 were cultivated and 7 were water areas; these 40 pinch points were mainly distributed along the corridors in the central part of the Jianghan Plain, with some pinch points in the ecological corridors in the northeastern part of the Jianghan Plain. The ecological corridors connecting the center to the southern part of the Jianghan Plain have a high priority for protection, and they are mostly oriented from northwest to southeast.

**Figure 11:**
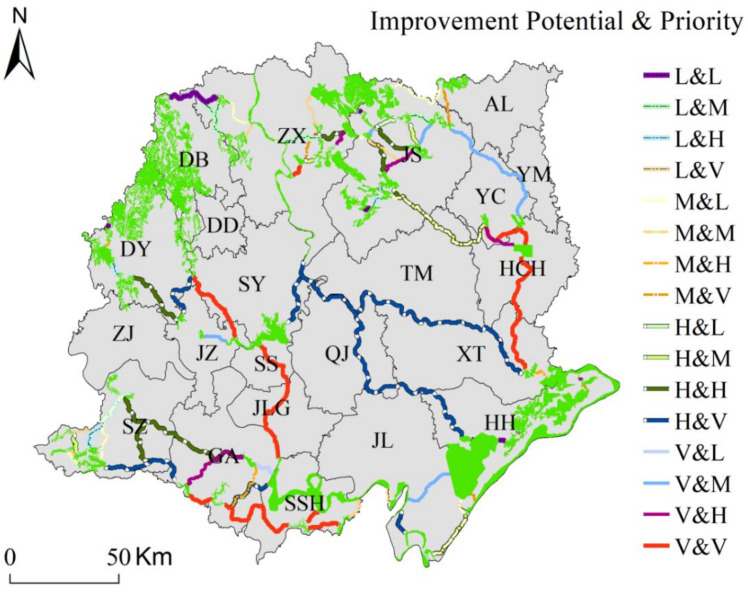
Composite map for improvement potential level and priority; L means low level; M means medium level; H means high level; V means very high level; L&L means the improvement potential level of the corridor is at low level and, at the same time, the priority level of this corridor is at a low level.

We overlaid the improvement potential level map and priority level map to create a composite map identifying the corridors with the most potential for improvement and the most conservation value (Figure 11). The result showed a total of 11 corridors with a very high level of improvement potential and priority. With the potential for connectivity enhancement, these important corridors mainly connect the central and southern ESs of the Jianghan Plain. The connected ecological reserves were as follows: Pengchang Heron Lake Wetland in Shayang (SY), Hubei Jingzhou Changhu National Wetland in Jinzhou (JZ), Mochou Lake, Hanjiang Soil and Water Conservation Ecological Protection Red Line in Zhongxiang (ZX), Hubei Jingzhou Changhu National Wetland in Shashi (SS), East and West Cha Lake in Yingcheng (YC), Hanbei River Wahoo National Aquatic Germplasm Resource Reserve in Yingcheng (YC), Hanbei River Wa’s Pelteobagrus fulvidraco National Aquatic Germplasm Resource Reserve in Hanchuan (HCH), Hanchuan Lake National Wetland Park, Hubei Yangtze river Swanzhou Baiji Dolphin National Nature Reserve in Shishou (SSH), Silt Lake, Niu Lang Lake, Huangtian Lake in Gongan (GA), Hubei Yangtze river Swanzhou Baiji Dolphin National Nature Reserve in Gongan (GA), Zhongdock Water Treatment Plant Water Containment Ecological Protection Red Line in Gongan (GA), Shangjin Lake National Aquatic Germplasm Resource Reserve in Shishou (SSH), Xiaogan Lao Guanghu National Wetland Nature Park in Yingcheng (YC).

## 4. Discussion

### 4.1. Discussion on ESP Construction

Jianghan Plain is located in a subtropical monsoon region, a highly populated area for both urban and agriculture development in central China. In terms of the problem-driven segment, considering the special ecological issues and geospatial differences of Jianghan, we selected a number of key metrics accordingly. For instance, the evaluation of biodiversity, water yield, and carbon storage were particularly meaningful for depicting the vital ecological space in Jianghan Plain, because of the severe biodiversity loss and documented water pollution under climate change and fast industrialization. In addition, we combined ecosystem service indicators and ecological sensitivity indicators when identifying the ecological sources. This ecological source selection framework is corresponding to the national standard of territorial spatial planning—to find the conservation places that are ecologically important, ecologically sensible, and ecologically fragile. We constructed a set of index systems in line with the regional characteristics. The construction of ecological resistance surface in the Jianghan Plain could be applied to similar areas that also have a monsoon climate background and the high-intensity human activity interference. Previous studies [93] only uniformly assign values to land-use types to determine the basic resistance surface, which is easy and low cost. However, this method is inaccurate because they did not comprehensively consider the unique geographical characteristic, such as the impact of topological factors and other invisible environmental factors, such as the influence of gas pollution or wastewater on the environment. This paper sets a peculiar ecological environment condition of the Jianghan Plain area and combines elevation, slope, land cover type, topological factors, distance to roads, distance to pollution sources to construct the basic resistance surface and offers a reference for choosing the resistance factor of the populated area.

Unlike using only the minimum cumulative resistance model and land use cover data when constructing ecological corridors that many ESP studies have completed, our study integrated transdisciplinary knowledge (circuit theory from physics, least-cost path analyses from GIS, and landscape ecology from ecology and geography), methodology, and multisource data to construct a holistic framework for designing ESP patterns. Moreover, another novelty of the framework lies in that we extracted the important ecological corridors, classified them according to their centrality, connectivity improvement potential, and priority (importance) and identified 40 sites with the highest improvement potential and highest protection needs. This meets the realistic needs of China’s territorial spatial planning and enables the use of feasible methods and available information to find sites for ecological conservation and ecological restoration. The interdisciplinary knowledge of landscape management and integrated methods, such as assessment of ecosystem services and ecological sensitivity, the least-cost path, circuit theory, and the centrality index, were used to construct ESPs and foster harmony between humanity and nature. Due to the great complexity that is conditioned by heterogeneous ecological and geographical contexts, no standard method has yet been reached.

Considering the different topography and regional economic development status of different regions, the demand for ecological services and ecological damage are different, which influence the selection of indicators for ecological quality evaluation, ecological sensibility evaluation, and resistance factors. For example, in the Karst area, to construct the basic resistance surface, researchers need to consider the mechanism for the rock desertification formation. When choosing indicators for constructing resistance surface, researcher could combine the regular indicators such as elevation, slope, land cover type, vegetation coverage, and particular indicators that could illustrate the Karst geography, such as lithology and soil thickness [102]. The logical framework of this research is in line with national needs, while the selection of indicators is not universal. Although an accurate evaluation of corridor efficiency remains challenging, the restoration measures targeting priority corridors are far more effective than those implemented in a random fashion. This article can serve as a reference for setting parameters and research frameworks for ecological security patterns in subtropical monsoonal and densely populated areas.

### 4.2. Discussion on Robustness of the Results

**Figure A1:**
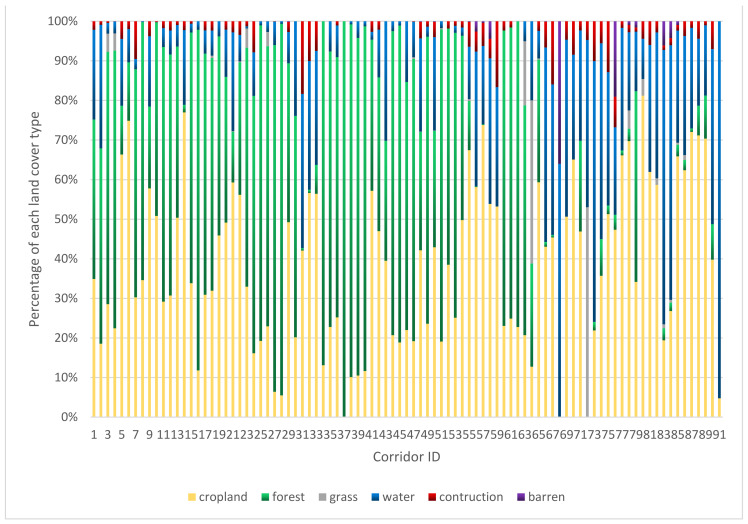
Land use structure of the ecological corridors.

**Figure A2:**
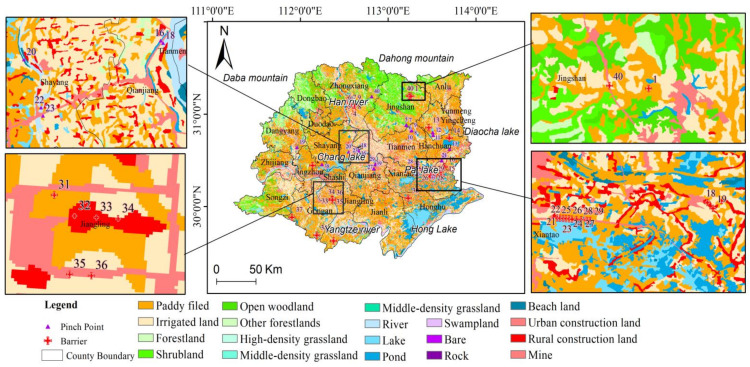
Top 40 pinch points and top 40 barriers in Jianghan Pl.

We demonstrated the robustness of the results from three aspects. First, the National Ecological Protection Red Line of Hubei Province was positioned within the ES patches that we identified. As China’s ecological protection red line delineates land where land-cover changes are not allowed, which involves a conflict of interest between ecological protection and local economic development, the area of the ecological protection red line delineated by the Department of Natural Resources of Hubei Province is relatively smaller than that of the land identified by the integrated approach in this study. Second, the wetlands surrounding lakes and rivers in the southern region and in the hills of the northwestern region are abundant with protected wildlife species [16], and the central Jianghan Plain, which occupies mostly cropland and built-up areas, has less protected wildlife. Third, to verify the feasibility of protecting the corridors, we analyzed the ecological land proportion by overlapping the land-cover map with corridors with a 1 km width. The results showed that 46.61% of corridors were cropland, 26.86% were woodland, and 20.12% were water bodies, which indicated that protecting the selected corridors in the real world is feasible. The land structure of each ecological corridor is presented in Appendix A (Figure A1). The information about the top 40 pinch points and top 40 barriers is presented in Appendix A (Figure A2, Table A2 & Table A3).

### 4.3. Discussion on Weakness of this Research

This research has two weaknesses that remain to be improved. First, the evaluation methods of ecological quality and ecological sensitivity in our research are commonly used by ecological evaluation studies on large scales. However, the quality of ecological evaluation methods and results could be further enhanced if we had more field data. For example, if we had water quality sample data, we would know the overall spatial pattern of water quality in the study area through remote sensing inversion. By doing so, we could distinguish the habitat quality and resistance values of the water area. Thus, improving assessment quality requires the improvement of evaluation methods and an increase in field data. Second, the parameterization (e.g., resistance value for land-cover types, the size of ESs, search radius for barrier analysis) was based on subjective determinates by referring to similar studies in Central China, which may introduce bias in the analysis results. With the diversification and enrichment of geographic data, these problems will be solved.

## 5. Conclusions

In the context of rapid urbanization, human beings are using land intensively, which will affect the sustainability of human society and jeopardize the quality of human life in the long run. Therefore, it is urgent to understand the current spatial distribution of ecological services and ecological sensitivity to construct EN patterns. We constructed an EN pattern in the Jianghan Plain and prioritized corridors by developing an integrated framework. Our results identified 48 ESs and 91 corridors on the Jianghan Plain. ESs were mainly distributed in the water area and wetlands in the north and southeastern Jianghan Plain, mainly located in Zhongxiang city, Dangyang city, Dongbao District of Jinmen Honghu Shishou city, and Songzi city. Ecological corridors could be divided into three sets based on their spatial pattern: one set of corridors was connected the ESs from central or north to south, one set connected the ES sites that were relatively parallel to latitudes in the north of the study area, and another set of ecological corridors were parallel to latitudes in the south. There were 11 priority corridors with a very high level of connectivity improvement potential and a very high level of conservation priority, occupying 16.15% of the total length. The corridors with high connectivity improvement potential and high priority were mainly located in the central and southern parts of the Jianghan Plain. These corridors should be well protected and restored.

This study identified corridor barriers and priorities by combining circuit theory with GIS spatial analyses, showed the spatial heterogeneity of ecological corridors, and identified the nodes needing further restoration and conservation. This study provides an efficient way to help local decision-makers in natural connectivity planning.

## Figures and Tables

**Table 1:** Data description.

Data Name	Data Source	Time	Units/Resolution
Depth to bedrock map of China	Scientific data [72]	2018	100 m × 100 m
Soil types	Harmonized World Soil Database version 1.2 (HWSD V1.2) [73]		1:1,000,000
Land-use/land cover data	Resource and Environment Science and Data Center [74]	2020	30 m × 30 m
Ecological red line of Hubei	Department of Natural Resource of Hubei Province	2020	1:250,000
Road	Baidu Map; Open Street Map	2020	1:250,000
Meteorological data	Meteorological Data Center of China Meteorological Administration [75]	2019	Daily
Digital elevation model	Shuttle Radar Topography Mission [76]	2008	30 m × 30 m
List of pollution factories in Hubei, 2017	Department of Ecological and Environment of Hubei Province; Ovitalmap.	2017	

**Table 2:** The standard for habitat sensibility score.

	Score	7	5	3	1
**Habitat Sensibility**	Distance to major road (m)	[0, 1000)	[1000, 2000)	[2000, 3000)	[3000, +∞)
	Distance to natural reserves (m)	[0, 3000)	[3000, 6000)	[6000, 9000)	[9000, +∞)
	land-cover type	Forestlands; water bodies; wetland	Grass; cropland	Barren	Urban or built-up
	NDVI	[0.7, 1]	[0.5, 0.7)	[0.3, 0.5)	[0, 0.3)

**Table 3:** The standard for water sensibility score.

	Score	7	5	3	1
**Water sensibility**	Distance to rivers, lakes, etc. (m)	[0, 500)	[500, 1000)	[1000, 1500)	[1500, +∞)
	Distance to wastewater sources or sewage treatment plant (m)	[0, 3000)	[3000, 6000)	[6000, 9000)	[9000, +∞)
	Distance to polluting air sources (m)	[0, 4000)	[4000, 8000)	[8000, 12000)	[12000, ∞)

**Table 4:** Resistance value criteria for different resistance types.

Resistance Types	Factors		Weight	Resistance Value
**Land Use and Land Cover**	Paddy filed		0.3	100
	Irrigated land			90
	Forestland			1
	Shrubland			1
	Open woodland			1
	Other forestlands			1
	High-density grassland			50
	Middle-density grassland			60
	Low-density grassland			70
	River			10
	Lake			10
	Pond			10
	Beach land			10
	Urban construction land			800
	Rural construction land			700
	Mine			1000
	Swampland			50
	Bare			50
	Rock			50
**Topological Factors**	Relief/m	[0, 25]	0.15	100
		(25, 50]		80
		(50, 75]		60
		(75, 100]		40
		(100, 239]		1
	Slope/°	[0, 8]	0.15	100
		(8, 15]		80
		(15, 25]		60
		(25, 35]		40
		(35, 47]		1
**Road**	Distance to road I (railroad, national highway, provincial highway)	[0, 200]	0.15	100
		(200, 400]		80
		(400, 800]		60
		(800, 1600]		40
		(1600, 3200]		20
		(3200, + ∞)		1
	Distance to road II (country highway)	[0, 150]	0.15	100
		(150, 250]		80
		(250, 450]		60
		(450, 800]		40
		(800, 1000]		20
		(1000, + ∞)		1
**Pollution Sources**	Distance to pollution sources (m)	[0, 2000)	0.1	100
		[2000, 4000)		80
		[6000, 8000)		60
		[8000, 10,000)		40
		[10,000, + ∞)		1

## Data Availability

Not applicable.

## Appendix A

**Table A1:** The explanation of the evaluation models and model parameters.

Evaluation Indicators	Evaluation Model	Model Parameters	Parameters Sources	Reference
Habitat Quality	Habitat Quality module in InVEST model	Distance between the habitat and threat sources	Calculated using Land use/cover map	Li et al. (2021) [83]
		Level of legal in each cell	Default value	
		Relative impact of each threat	Assignment values according to previous studies	
		Relative sensitivity of each habitat type to threat		
Carbon Storage	Carbon storage and sequestration module in InVEST model	Carbon pools		Tang et al. (2020) [103]
		Land use/cover	Derived from Land use/cover map	
Water Yield	Water yield module in InVEST model	Land use/cover		Yang et al. (2019) [92]
		Precipitation	Calculated using Meteorological data	
		Average annual reference evapotranspiration		
		Root restricting layer depth	Calculated using Soil data	
		Plant available water content		
		Z parameter	Assignment values according to previous studies	
		Watersheds/ sub watersheds	Calculated using DEM	
Soil Erosion Sensitivity	CSLE model	Slope length L/slope steepness S		Liu et al. (2020); Gao et al. (2021) [97,104]
		Rainfall erosivity R	Calculated using Meteorological Data	
		Soil erodibility K	Calculated using Soil data	
		Cover management C/engineering practices P/tillage practices T	Assignment values according to previous studies	
Water Sensitivity	The distance to water body	Classification of distance to water body	Calculated using land-use and land cover map	Yimin et al. (2018); Xiao et al. (2020) [93,94]
	Distance to wastewater sources or sewage treatment plant (m)	Classification of distance to wastewater sources or sewage treatment plant	Calculated using wastewater sources map and sewage treatment plant distribution map	
	Distance to polluting air sources(m)	Classification of distance to polluting air sources	Calculated using polluting air sources map	
Habitat Sensitivity	The distance to major roads	Classification of distance to major roads	Calculated using transportation planning map	Rayfield et al. (2010); Yimin et al. (2018) [94,105]
	The distance to nature reserves	Classification of distance to nature reserves	Calculated using nature reserve distribution map	
	Land use types	Classification of Land use types	Reclassified using land use/cover map	
	NDVI	Classification of NDVI value	Reclassified using NDVI	

**Table A2:** Information about top 40 pinch points.

Rank	Land Cover Type	Latitude	Longitude	County
1	Lake	112.63	31.11	Zhongxiang
2	Lake	112.62	31.10	Zhongxiang
3	Paddy filed	112.62	31.10	Zhongxiang
4	Paddy filed	112.62	31.09	Zhongxiang
5	Shrubland	113.21	30.89	Jingshan
6	Shrubland	113.21	30.88	Jingshan
7	Shrubland	113.21	30.88	Jingshan
8	Pond	113.27	30.83	Tianmen
9	open woodland	113.27	30.83	Tianmen
10	open woodland	113.26	30.82	Tianmen
11	River	113.51	30.80	Yingcheng
12	River	113.51	30.77	Yingcheng
13	Pond	113.70	30.62	Hanchuan
14	River	113.64	30.57	Hanchuan
15	River	113.64	30.55	Hanchuan
16	Pond	112.66	30.61	Qianjiang
17	River	113.64	30.54	Hanchuan
18	River	112.66	30.61	Qianjiang
19	Beach land	111.96	30.64	Dangyang
20	River	112.55	30.59	Shayang
21	River	113.61	30.50	Hanchuan
22	River	112.57	30.56	Shayang
23	Irrigated land	112.57	30.55	Shayang
24	Paddy filed	113.64	30.43	Hanchuan
25	Irrigated land	113.63	30.43	Hanchuan
26	River	113.34	30.43	Xiantao
27	River	112.86	30.46	Qianjiang
28	River	113.26	30.43	Xiantao
29	Beach land	112.86	30.46	Qianjiang
30	Paddy filed	113.61	30.40	Xiantao
31	Paddy filed	113.60	30.40	Xiantao
32	River	113.39	30.41	Xiantao
33	River	113.47	30.39	Xiantao
34	River	113.47	30.39	Xiantao
35	River	113.47	30.39	Xiantao
36	River	113.45	30.39	Tianmen
37	River	113.47	30.39	Xiantao
38	Lake	112.25	30.46	Jingzhou
39	River	112.84	30.41	Qianjiang
40	Irrigated land	112.81	31.19	Zhongxiang

**Table A3:** Information about top 40 barriers.

Rank	Land Cover Type	Latitude	Longitude	County
1	Paddy filed	113.26	31.20	Jingshan
2	Rural construction land	112.61	31.10	Zhongxiang
3	Rural construction land	112.61	31.10	Zhongxiang
4	Paddy filed	112.61	31.10	Zhongxiang
5	Mine	112.62	31.10	Zhongxiang
6	Paddy filed	112.61	31.10	Zhongxiang
7	Rural construction land	112.61	31.10	Zhongxiang
8	Rural construction land	112.61	31.10	Zhongxiang
9	Rural construction land	112.61	31.10	Zhongxiang
10	Rural construction land	112.61	31.10	Zhongxiang
11	Rural construction land	112.61	31.10	Zhongxiang
12	Paddy filed	112.62	31.10	Zhongxiang
13	Urban construction land	113.47	30.86	Yingcheng
14	Paddy filed	113.70	30.74	Hanchuan
15	Rural construction land	113.64	30.43	Hanchuan
16	Rural construction land	113.64	30.43	Hanchuan
17	Paddy filed	112.14	30.45	Jingzhou
18	Rural construction land	113.60	30.34	Xiantao
19	Rural construction land	113.60	30.34	Xiantao
20	Mine	113.49	30.33	Xiantao
21	Mine	113.50	30.33	Xiantao
22	Mine	113.50	30.33	Xiantao
23	Mine	113.50	30.33	Xiantao
24	Mine	113.50	30.33	Xiantao
25	Mine	113.50	30.33	Xiantao
26	Mine	113.51	30.33	Xiantao
27	Mine	113.51	30.33	Xiantao
28	Mine	113.52	30.33	Xiantao
29	Mine	113.52	30.33	Xiantao
30	Paddy filed	113.23	30.09	Honghu
31	Paddy filed	112.36	30.08	Jiangling
32	Rural construction land	112.37	30.08	Jiangling
33	Rural construction land	112.37	30.08	Jiangling
34	Rural construction land	112.37	30.08	Jiangling
35	Mine	112.36	30.07	Jiangling
36	Mine	112.37	30.07	Jiangling
37	Rural construction land	111.91	29.88	Gongan
38	Paddy filed	112.19	29.69	Gongan
39	Paddy filed	112.38	29.63	Shishou
40	Mine	113.24	31.20	Jingshan
